# Statistical inference on nonparametric regression model with approximation of Fourier series function: Estimation and hypothesis testing

**DOI:** 10.1016/j.mex.2024.102922

**Published:** 2024-08-16

**Authors:** Mustain Ramli, I Nyoman Budiantara, Vita Ratnasari

**Affiliations:** Department of Statistics, Faculty of Science and Data Analytics, Institut Teknologi Sepuluh Nopember, Kampus ITS-Sukolilo, Surabaya 60111, Indonesia

**Keywords:** Nonparametric regression, Fourier series, Statistical inference, Likelihood ratio test, Life expectancy data, Fourier Series Function

## Abstract

Nonparametric regression is an approximation method in regression analysis that is not constrained by the assumption of knowing the regression curve. One of the functions to approximate the curve is a Fourier series function. The nonparametric regression model with approximation of a Fourier series function has been widely discussed by several researchers. However, discussions on statistical inference, particularly in partial hypothesis testing, has not been carried out previously. Therefore, the purpose of this research is to discuss the statistical inference on nonparametric regression model with approximation of a Fourier series function. The discussion includes parameter and model estimations, simultaneous and partial hypotheses testing. In the application, we use life expectancy data from East Java Province during 2022. Based on data analysis, we obtain a model estimation with an R-square value of 96.24 %. At a 5 % significance level, the parameters simultaneously have a significant influence on the model. Partially, four parameters are not significant. However, overall, the predictor variables significantly influence the life expectancy data.

• The Fourier series function used is a Fourier series function introduced by Bilodeau (1992).
• The model estimation is obtained by selecting the optimal number of oscillation parameters.
• The statistical test is obtained using the LRT method.

## Graphical abstract

**Figure:**
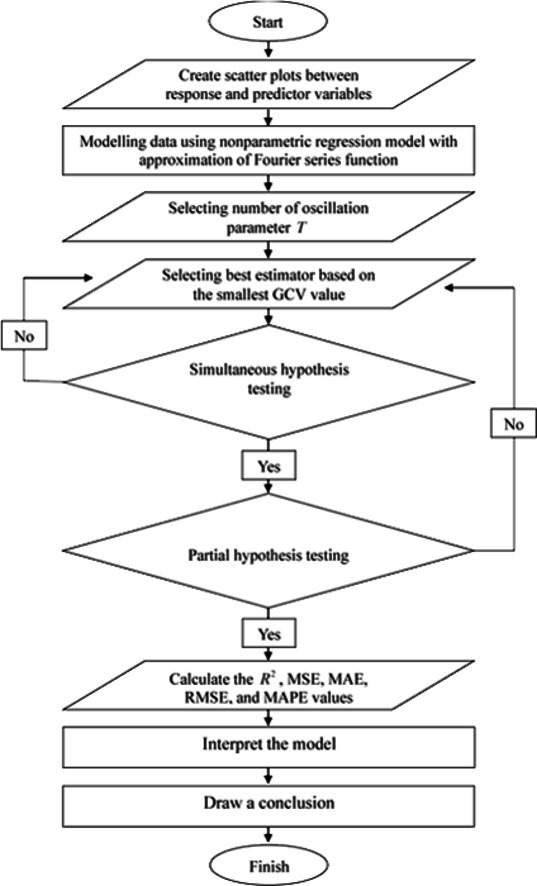

Specifications table

**Table:** 

Subject area:	Mathematics and Statistics
More specific subject area:	Statistics; Nonparametric Regression
Name of your method:	Fourier Series Function
Name and reference of original method:	Fourier series function developed by Bilodeau (1992), M. Bilodeau, Fourier smoother and additive models, The Canadian Journal of Statistics 20 (1992) 257–269. https://doi.org/10.2307/3315313
Resource availability:	Life expectancy data and its predictor variables can be accessed through the website of the Badan Pusat Statistik (BPS) of East Java Province at https://jatim.bps.go.id/

## Background

Nonparametric regression is an approximation method in regression analysis that is not constrained by the assumption of knowing the regression curve. Moreover, nonparametric regression offers high flexibility, as the regression curve can be adapted to the local nature of the data [1]. However, a nonparametric regression curve cannot be determined arbitrarily without information from the data, such as examining data patterns based on a scatterplot [[2], [3]]. In nonparametric regression, there are several estimator approaches for the regression curve, such as Spline function [[4], [5], [6]], Kernel function [[7], [8], [9]], Fourier series function [[10], [11], [12], [13]], and many others. Among these approaches, Fourier series function is particularly intriguing to many researchers in the field of nonparametric regression due to its ability to handle data that exhibits repeated patterns at certain interval. According to Srivani et al. and Steinerberger, Fourier series is a trigonometric polynomial function that combines cos and sin functions [14,15]. However, Bilodeau introduced a new Fourier series function for a nonparametric regression model, which includes only the cos function and incorporates a trend to the function [10]. The Fourier series estimator introduced by Bilodeau has been widely adopted and further developed by numerous researchers for estimating the nonparametric regression curve [see, 13,[16], [17], [18], [19]]. In the application, the Fourier series estimator introduced by Bilodeau has been use for modelling various different data [[16], [17],[19], [20], [21]].

In regression analysis, statistical inference plays a crucial role in obtaining the best model estimates. Generally, statistical inference in regression analysis is divided into two main areas: estimation and hypothesis testing. Estimation in regression analysis involves estimating the parameters in the model. These parameter estimates provide a model estimation that is useful for drawing conclusions about the response variable based on the given predictor variables. Moreover, hypothesis testing in regression analysis aims to determine whether the parameters significantly influence the model. Generally, hypothesis testing in regression analysis is divided into two categories: simultaneous and partial hypotheses testing. Simultaneous hypothesis testing aims to determine the combined influence of all parameters on the model. If this testing indicates that all parameters simultaneously have a significant influence, then partial hypothesis testing is performed to identify which specific parameters influence the model. In the field of regression analysis, statistical inference has been widely discussed by several researchers [[22], [23], [24]].

The discussion related to statistical inference in nonparametric regression model with approximation of a Fourier series function has so far primarily focused on parameter and model estimation [10,12,[16], [17], [18],[20], [21]]. Ramli, et al. is the only researcher who discussed hypothesis testing in nonparametric regression model with approximation of a Fourier series function, focusing on simultaneous hypothesis testing [19]. Meanwhile, partial hypothesis testing in this model has not been carried out. The significant role of statistical inference in regression analysis, especially in nonparametric regression model with approximation of a Fourier series function makes this an interesting topic for discussion in this research. Although several aspects of statistical inference in this model, such as estimation and simultaneous hypothesis testing have been previously discussed both theoretically and practically, this research will only briefly discuss upon these areas. Therefore, the primary focus will be on the theoretical development of partial hypothesis testing, which has not been carried out previously. Furthermore, to apply statistical inference in nonparametric regression model with approximation of a Fourier series function, we use life expectancy data from 38 districts and cities in East Java Province during 2022.

## Method details

### The model and its estimation

In this section, we provide a general description of nonparametric regression model with approximation of a Fourier series function. The Fourier series function we utilize follows the formulation introduced by Bilodeau [10]. Parameter estimation in the nonparametric regression model with approximation of a Fourier series function is conducted using the Ordinary Least Squares (OLS) method, a technique employed by several previous researchers. Additionally, the selection of optimal parameter estimates for the model employs the Generalized Cross Validation (GCV) method.

#### The nonparametric regression model with approximation of a Fourier series function

Suppose $y_{i}$ is the response variable and $x_{i1},x_{i2},...,x_{ip}$ are the p predictor variables, where i=1,2,...,n. Assuming the relationship between the response and predictor variables follows the nonparametric regression model as describe in Eq. (1).

(1) $$y_{i}=g\left(x_{i1},x_{i2},...,x_{ip}\right)+\varepsilon_{i},\varepsilon_{i}∼N\left(0,\sigma^{2}\right),$$

Assuming between the predictor variables are not correlated, the nonparametric regression model in Eq. (1) can be written as an additive model.

(2) $$y_{i}=\sum_{j=1}^{p}g\left(x_{ij}\right)+\varepsilon_{i}.$$

Since $g(x_{ij})$ is the nonparametric regression curve which we assume as an unknown function, $g(x_{ij})$ can be approximated using one of the estimators in nonparametric regression model, specifically the Fourier series function [10], the Fourier series function is given in Eq. (3).

(3) $$g\left(x_{ij}\right)=\frac{\mu_{j}}{2}+\beta_{j}x_{ij}+\sum_{t=1}^{T}\delta_{tj}\cos\left(tx_{ij}\right),$$

where $\mu_{j}$ is the constant parameter (representing the baseline level), $\beta_{j}$ is the trend parameter (representing a linear trend into the function), and $\delta_{tj}$ is the parameter for the cos terms (adding periodic component to the function), for j=1,2,...,p and t=1,2,...,T with T is the oscillation parameter.

Furthermore, since $g(x_{ij})$ is approximated with a Fourier series function then by substituting Eq. (3) into Eq. (2), we obtain the nonparametric regression model with approximation of a Fourier series function as follows.

(4) $$y_{i}=\sum_{j=1}^{p}\left(\frac{\mu_{j}}{2}+\beta_{j}x_{ij}+\sum_{t=1}^{T}\delta_{tj}\cos\left(tx_{ij}\right)\right)+\varepsilon_{i}.$$

The nonparametric regression model with approximation of a Fourier series function in Eq. (4) can be formulated in matrix and vector form as in Eq. (5), where the description holds for i=1,2,...,n,j=1,2,...,p and t=1,2,...,T [19].

(5) $$y=X\delta +\varepsilon ,\varepsilon ∼N\left(0,I\sigma^{2}\right),$$

where $y={\left[\begin{array}{cccc} y_{1} & y_{2} & \cdots & y_{n} \end{array}\right]}^{\prime }$ is a vector containing the response variable with the size of n×1, $X=[\begin{array}{ccccccc} X_{1}\left(T\right) & \vdots & X_{2}\left(T\right) & \vdots & \cdots & \vdots & X_{p}\left(T\right) \end{array}]$ is a matrix containing the predictor variables with a Fourier series function with the size of $n\times p\left(T+2\right),\;\delta ={\left[\begin{array}{ccccccc} \delta_{1} & \vdots & \delta_{2} & \vdots & \cdots & \vdots & \delta_{p} \end{array}\right]}^{\prime }$ is a vector containing the parameters in the model with the size of p(T+2)×1, and $\varepsilon ={\left[\begin{array}{cccc} \varepsilon_{1} & \varepsilon_{2} & \cdots & \varepsilon_{n} \end{array}\right]}^{\prime }$ is a vector containing the error in the model with the size of n×1. The elements of matrices $X_{1}\left(T\right),X_{2}\left(T\right),...,X_{p}\left(T\right)$ and vectors $\delta_{1},\delta_{2},...,\delta_{p}$ are given as follows.

$$X_{j}\left(T\right)=\left[\begin{array}{cccccc} \frac{1}{2} & x_{1j} & \cos\left(x_{1j}\right) & \cos\left(2x_{1j}\right) & \cdots & \cos\left(Tx_{1j}\right)\\ \frac{1}{2} & x_{2j} & \cos\left(x_{2j}\right) & \cos\left(2x_{2j}\right) & \cdots & \cos\left(Tx_{2j}\right)\\ \vdots & \vdots & \vdots & \vdots & \ddots & \vdots \\ \frac{1}{2} & x_{nj} & \cos\left(x_{nj}\right) & \cos\left(2x_{nj}\right) & \cdots & \cos\left(Tx_{nj}\right) \end{array}\right],\;\text{for}\;j=1,2,...,p$$

$$\delta_{j}={\left[\begin{array}{cccccc} \mu_{j} & \beta_{j} & \delta_{1j} & \delta_{2j} & \cdots & \delta_{Tj} \end{array}\right]}^{\prime },\;\text{for}\;j=1,2,...,p$$

#### Parameter and model estimations

In regression analysis, obtaining model estimates is equivalent to estimating the parameters in the model. In nonparametric regression model with approximation of a Fourier series function, parameter estimates can be obtained using several estimation methods, such as Penalized Least Square (PLS) method [10,12,18,20] and OLS method [[16], [17],^19^,^21^]. The choice between these two methods depends on the form of the curve $g(x_{ij})$. If the curve $g(x_{ij})$ is assumed to be a smooth function, then the Partial Least Squares (PLS) method is utilized. On the other hand, if we simply aim to obtain parameter estimates without assuming that the curve $g(x_{ij})$ must be smooth, we can use the OLS method. In general, the OLS method is a classic approach in regression analysis for obtaining parameter estimates by minimizing the sum of squared errors. In this research, we employ the OLS method for parameter estimation. Therefore, based on the nonparametric regression model with approximation of a Fourier series function in Eq. (5), the parameter estimation using the OLS method is obtained as follows [[16], [17],^19^,^21^].

(6) $$\hat{\delta }=\arg\min\left\{\varepsilon^{\prime }\varepsilon \right\}\underset{\mu \in R^{p\left(T+2\right)}}{=\arg\min}\left\{{\left(y-X\delta \right)}^{\prime }\left(y-X\delta \right)\right\}={\left(X^{\prime }X\right)}^{-1}X^{\prime }y,$$

where $\hat{\delta }$ in Eq. (6) is the parameter estimation for δ in Eq. (5). Furthermore, based on $\hat{\delta }$ in Eq. (6), we obtain the estimation of the nonparametric regression model with approximation of a Fourier series function as follows.

(7) $$\hat{y}=X\hat{\delta }=X{\left(X^{\prime }X\right)}^{-1}X^{\prime }y=\mathrm{Uy},$$

where $U=X{\left(X^{\prime }X\right)}^{-1}X^{\prime }$ and $\hat{y}$ in Eq. (7) is the model estimation.

#### Model selection

In contrast to parametric regression models, where estimates are obtained directly from the parameters without the need for selecting an optimal model, nonparametric regression models require the selection of an optimal model estimate. Despite using approaches such as Spline, Kernel, or Fourier series functions to approximate the regression curve, there is always at least one parameter that necessitates choosing the optimal model estimate. The parameters in question are the knot points in a Spline function [[4], [5], [6]], the bandwidth in a Kernel function [[7], [8], [9]], and the oscillation parameters in a Fourier series function [16,19,21]. Therefore, a method is needed to select the optimal model. One such method is the GCV method. The GCV method was First introduced by Craven and Wahba [25]. This method has been widely used in nonparametric regression models for optimal model selection. Selecting an optimal nonparametric regression model with approximation of a Fourier series function involves choosing the optimal oscillation parameters based on the minimum GCV value. The GCV function for selecting optimal oscillation parameters is given in Eq. (8) [16,19,21].

(8) $$\text{GCV}\left(T_{\text{Optimum}}\right)=\min\left\{\frac{n^{-1}y^{\prime }{\left(I-U\right)}^{\prime }\left(I-U\right)y}{{\left(n^{-1}trace\left[I-U\right]\right)}^{2}}\right\}.$$

### Simultaneous hypothesis testing

Simultaneous hypothesis testing is used to evaluate the influence of all parameters simultaneously in a regression model. This type of testing, particularly in nonparametric regression model with approximation of a Fourier series function was developed by Ramli et al. [19]. Therefore, explanations related to simultaneous hypothesis testing are provided briefly to support the application of the data in this research. The first step in simultaneous hypothesis testing is to formulate the hypothesis form. Based on the nonparametric regression model with approximation of a Fourier series function in Eq. (4), we can define the hypothesis form for simultaneous hypothesis testing as follows.

(9) $$\begin{array}{ccc} & & H_{0}:\mu_{1}=\mu_{2}=...=\mu_{p}=\beta_{1}=\beta_{2}=...=\beta_{p}=\delta_{11}=\delta_{21}=...=\delta_{T1}=...=\delta_{1p}=\delta_{2p}=...=\delta_{Tp}=0\\ & & H_{1}:\text{There}\;\text{is}\;\text{minimum}\;\text{one}\;\text{of}\;\mu_{j}\neq 0,\beta_{j}\neq 0,\delta_{tj}\neq 0,\;\text{for}\;j=1,2,...,p\;\text{and}\;t=1,2,...,T \end{array}$$

The hypothesis form given in Eq. (9) is a specific hypothesis form used to evaluate the influence of all parameters on the model without testing the magnitude of their influence. Furthermore, to test the hypothesis form in Eq. (9), it is necessary to obtain a formula for the statistical test. One method to obtain a statistical test is the Likelihood Ratio Test (LRT) method. According to Ramli et al., using the LRT method, the formulation of the statistical test for simultaneous hypothesis testing with the hypothesis form given in Eq. (9) is as follows [19].

(10) $$\Lambda =\frac{\frac{{\left(X\hat{\delta }\right)}^{\prime }y}{p\left(T+1\right)+1}}{\frac{{\left(y-X\hat{\delta }\right)}^{\prime }\left(y-X\hat{\delta }\right)}{n-\left(p\left(T+1\right)+1\right)}},$$

where the statistical test of Λ in Eq. (10) is the statistical test for simultaneous hypothesis testing in nonparametric regression model with approximation of a Fourier series function. Furthermore, according to Ramli et al. [19], the statistical test of Λ follows an F distribution with degrees of freedom p(T+1)+1 and n−(p(T+1)+1). Given a significance level α for testing the hypothesis in Eq. (9), the null hypothesis will be rejected at significance level α if and only if the statistic value of Λ is greater than the statistic value of $F_{\left(\alpha ,p(T+1)+1,n-(p(T+1)+1)\right)}$, where $F_{\left(\alpha ,p(T+1)+1,n-(p(T+1)+1)\right)}$ denotes the statistic value of F distribution with significance level α and degrees of freedom p(T+1)+1 and n−(p(T+1)+1).

### Partial hypothesis testing

In this section, we discuss partial hypothesis testing for parameters in nonparametric regression model with approximation of a Fourier series function. The partial hypothesis testing continues from the simultaneous hypothesis testing explained in the previous section. Following the same framework, the formula of the statistical test for partial hypothesis testing is derived using the LRT method. Furthermore, we will provide a more detailed discussion on the distribution of the statistical test and the rejection region of the null hypothesis. The initial step in partial hypothesis testing involves formulating the hypothesis form. Based on the nonparametric regression model with approximation of a Fourier series function described in Eq. (4), we can formulate the hypotheses form for partial hypothesis testing as presented in Table 1 below.

**Table 1:** Partial hypotheses form.

Null Hypothesis	Alternative Hypothesis	Null Hypothesis	Alternative Hypothesis	Null Hypothesis	Alternative Hypothesis
$H_{0}:\mu_{1}=0$	$H_{1}:\mu_{1}\neq 0$	$H_{0}:\beta_{p}=0$	$H_{1}:\beta_{p}\neq 0$	⋮	⋮
$H_{0}:\mu_{2}=0$	$H_{1}:\mu_{2}\neq 0$	$H_{0}:\delta_{11}=0$	$H_{1}:\delta_{11}\neq 0$	$H_{0}:\delta_{2p}=0$	$H_{1}:\delta_{2p}\neq 0$
⋮	⋮	$H_{0}:\delta_{12}=0$	$H_{1}:\delta_{12}\neq 0$	⋮	⋮
$H_{0}:\mu_{p}=0$	$H_{1}:\mu_{p}\neq 0$	⋮	⋮	$H_{0}:\delta_{T1}=0$	$H_{1}:\delta_{T1}\neq 0$
$H_{0}:\beta_{1}=0$	$H_{1}:\beta_{1}\neq 0$	$H_{0}:\delta_{1p}=0$	$H_{1}:\delta_{1p}\neq 0$	$H_{0}:\delta_{T2}=0$	$H_{1}:\delta_{T2}\neq 0$
$H_{0}:\beta_{2}=0$	$H_{1}:\beta_{2}\neq 0$	$H_{0}:\delta_{21}=0$	$H_{1}:\delta_{21}\neq 0$	⋮	⋮
⋮	⋮	$H_{0}:\delta_{22}=0$	$H_{1}:\delta_{22}\neq 0$	$H_{0}:\delta_{Tp}=0$	$H_{1}:\delta_{Tp}\neq 0$

Table 1 presents a partial hypothesis formulation for all parameters in nonparametric regression model with approximation of a Fourier series function. The number of hypothesis formulations in Table 1 corresponds to the number of parameters in the model. Consequently, testing these hypotheses individually is time-consuming and involves lengthy mathematical elaboration. To address this, we combine the hypotheses in Table 1 into a simpler form to obtain the statistical test, its distribution, and the rejection region for the null hypothesis. This is achieved by formulating the hypotheses in Table 1 into the following vector form.

(11) $$H_{0}:a_{l}\delta =0\;\text{versus}\;H_{1}:a_{l}\delta \neq 0$$

where $a_{l}$ is a row vector with the $l^{th}$ column is 1 and otherwise is 0 with l=1,2,...,L, where L is the number of rows in vector δ, defined as L=p(T+2).

To obtain the formula for the statistical test in partial hypothesis testing, where the hypothesis form is given in Eq. (11), we use the LRT method. The LRT method has been widely used in hypothesis testing across various regression models [see, 19,[26], [27], [28]]. The main concept of the LRT method is to compare the likelihood function of the model under the null hypothesis with the likelihood function of the model under the alternative hypothesis. Based on the nonparametric regression model in Eq. (2), where $\varepsilon_{i}$ is known to be normally distributed with mean 0 and variance $\sigma^{2}$, the likelihood function is obtained as follows.

(12) $$L\left(\varepsilon_{1},\varepsilon_{2},...,\varepsilon_{n}\right)=\prod_{i=1}^{n}\frac{1}{\sqrt{2\pi \sigma^{2}}}\exp\left(-\frac{\varepsilon_{i}^{2}}{2\sigma^{2}}\right)={\left(2\pi \sigma^{2}\right)}^{-\frac{n}{2}}\exp\left(-\frac{1}{2\sigma^{2}}\sum_{i=1}^{n}\varepsilon_{i}^{2}\right).$$

Furthermore, the likelihood function in Eq. (12) can be written as the following vector.

(13) $$L\left(\varepsilon \right)={\left(2\pi \sigma^{2}\right)}^{-\frac{n}{2}}\exp\left(-\frac{1}{2\sigma^{2}}\varepsilon^{\prime }\varepsilon \right).$$

Since the nonparametric regression curve $g(x_{ij})$ in Eq. (2) is approximated by the Fourier series function in Eq. (3), it follows from Eq. (5) that y=Xδ+ε. Therefore, we obtain ε=y−Xδ. Since ε=y−Xδ, the likelihood function in Eq. (13) can be written as follows.

(14) $$L\left(\delta ,\sigma^{2}\right)={\left(2\pi \sigma^{2}\right)}^{-\frac{n}{2}}\exp\left(-\frac{1}{2\sigma^{2}}{\left(y-X\delta \right)}^{\prime }\left(y-X\delta \right)\right),$$

where the likelihood function in Eq. (14) is the likelihood function for $y∼N(X\delta ,I\sigma^{2})$.

Furthermore, let ω be the parameter space under the null hypothesis and Ω be the parameter space under the hypothesis. Therefore, based on the hypothesis form in Eq. (11) and the likelihood function in Eq. (14), the parameter spaces ω and Ω can be determined as follows.

(15) $$\omega =\left\{\delta_{\omega }={\left[\begin{array}{ccccccc} \delta_{1} & \vdots & \delta_{2} & \vdots & \cdots & \vdots & \delta_{p} \end{array}\right]}^{\prime },\sigma_{\omega }^{2}|a_{l}\delta_{\omega }=0\right\}.$$

(16) $$\Omega =\left\{\delta_{\Omega }={\left[\begin{array}{ccccccc} \delta_{1} & \vdots & \delta_{2} & \vdots & \cdots & \vdots & \delta_{p} \end{array}\right]}^{\prime },\sigma_{\Omega }^{2}\right\}.$$

Based on the likelihood function in Eq. (14) and the parameter space under the null hypothesis (ω) in Eq. (15), the likelihood function under the parameter space ω is obtained as follows.

(17) $$L\left(\omega \right)={\left(2\pi \sigma_{\omega }^{2}\right)}^{-\frac{n}{2}}\exp\left(-\frac{1}{2\sigma_{\omega }^{2}}{\left(y-X\delta_{\omega }\right)}^{\prime }\left(y-X\delta_{\omega }\right)\right).$$

where the likelihood function in Eq. (17) has a condition that $a_{l}\delta_{\omega }=0$. Similarly, based on the likelihood function in Eq. (14) and the parameter space under the hypothesis (Ω) as defined in Eq. (16), the likelihood function under the parameter space Ω is obtained as follows.

(18) $$L\left(\Omega \right)={\left(2\pi \sigma_{\Omega }^{2}\right)}^{-\frac{n}{2}}\exp\left(-\frac{1}{2\sigma_{\Omega }^{2}}{\left(y-X\delta_{\Omega }\right)}^{\prime }\left(y-X\delta_{\Omega }\right)\right).$$

#### The statistical test

The statistical test for partial hypothesis testing with the hypothesis form in Eq. (11) is obtained using the LRT method. Based on the LRT method, we obtain a likelihood ratio by comparing the maximum likelihood function under parameter space ω and the maximum likelihood function under parameter space Ω. Therefore, based on the likelihood ratio, we derive a formula for the statistical test. Furthermore, the statistical test is obtained based on Theorem 1. However, before presenting Theorem 1, we first obtain the likelihood ratio using the following equation.

(19) $$\begin{array}{cc} w\left(x_{i1},x_{i2},...,x_{ip};y_{i}\right)=\frac{L\left(\hat{\omega }\right)}{L\left(\hat{\Omega }\right)}, & 0<w\leq 1 \end{array},$$

where w in Eq. (19) is the likelihood ratio, $L(\hat{\omega })$ is the maximum likelihood under the parameter space of ω, and $L(\hat{\Omega })$ is the maximum likelihood under the parameter space of Ω. According to Ramli et al., if Ω is the parameter space under the hypothesis as given in Eq. (16) with the likelihood function under the parameter space Ω is provided in Eq. (18), then the maximum likelihood under the parameter space Ω is obtained as follows [19].

(20) $$L\left(\hat{\Omega }\right)={\left(2\pi {\hat{\sigma }}_{\Omega }^{2}\right)}^{-\frac{n}{2}}\exp\left(-\frac{n}{2}\right),$$

where $L(\hat{\Omega })$ in Eq. (20) is the maximum likelihood under the parameter space Ω, with ${\hat{\sigma }}_{\Omega }^{2}=\frac{1}{n}{\left(y-X{\hat{\delta }}_{\Omega }\right)}^{\prime }\left(y-X{\hat{\delta }}_{\Omega }\right)$ is the variance under the parameter space Ω and ${\hat{\delta }}_{\Omega }={\left(X^{\prime }X\right)}^{-1}X^{\prime }y$ is the parameter under the parameter space Ω.

Furthermore, for $L(\hat{\omega })$ can be obtained by estimating the parameters within the parameter space ω, namely $\delta_{\omega }$ and $\sigma_{\omega }^{2}$. Since the parameter space ω has a specific constraint for the parameter $\delta_{\omega }$, where $a_{l}\delta_{\omega }=0$. Therefore, we can estimate $\delta_{\omega }$ using the Lagrange Multiplier (LM) method, which is commonly applied when parameters have specific constraints. This method is extensively discussed in the context of general linear hypotheses in parametric regression, as explained in Searle [29]. Based on the likelihood function under the parameter space ω in Eq. (17) with the parameter space ω defined in Eq. (15), the LM function is given as follows.

(21) $$F\left(\delta_{\omega },\theta \right)=M\left(\delta_{\omega }\right)+2\theta a_{l}\delta_{\omega },$$

where $M\left(\delta_{\omega }\right)={\left(y-X\delta_{\omega }\right)}^{\prime }\left(y-X\delta_{\omega }\right)=y^{\prime }y-2{\delta^{\prime }}_{\omega }X^{\prime }y+{\delta^{\prime }}_{\omega }X^{\prime }X\delta_{\omega }$.

Since the LM function in Eq. (21) contains two parameters, namely $\delta_{\omega }$ dan θ. Therefore, the estimation of parameters $\delta_{\omega }$ dan θ are obtained by taking the partial derivatives OF $F(\delta_{\omega },\theta )$ with respect to $\delta_{\omega }$ and θ, and then setting these derivatives equal to 0. The estimation of parameter is obtained as follows.

(22) $$\frac{\partial F\left(\delta_{\omega },\theta \right)}{\partial \delta_{\omega }}=\frac{y^{\prime }y-2{\delta^{\prime }}_{\omega }X^{\prime }y+{\delta^{\prime }}_{\omega }X^{\prime }X\delta_{\omega }+2\theta a_{l}\delta_{\omega }}{\partial \delta_{\omega }}=-2X^{\prime }y+2X^{\prime }X\delta_{\omega }+2\theta {a^{\prime }}_{l}=0.$$

Based on Eq. (22), we obtain the estimation of $\delta_{\omega }$ as follows.

(23) $${\hat{\delta }}_{\omega }={\left(X^{\prime }X\right)}^{-1}\left(X^{\prime }y-{a^{\prime }}_{l}\theta \right).$$

Furthermore, for the estimation of θ is obtained as follows.

(24) $$\frac{\partial F\left(\delta_{\omega },\theta \right)}{\partial \theta }=\frac{y^{\prime }y-2{\delta^{\prime }}_{\omega }X^{\prime }y+{\delta^{\prime }}_{\omega }X^{\prime }X\delta_{\omega }+2\theta a_{l}\delta_{\omega }}{\partial \theta }=2a_{l}\delta_{\omega }=0.$$

Based on Eq. (24), since $\delta_{\omega }$ is estimated by ${\hat{\delta }}_{\omega }$ in Eq. (23) then the estimation of θ is obtained as follows.

(25) $$\hat{\theta }={\left(a_{l}{\left(X^{\prime }X\right)}^{-1}{a^{\prime }}_{l}\right)}^{-1}a_{l}{\left(X^{\prime }X\right)}^{-1}X^{\prime }y.$$

Since ${\hat{\delta }}_{\omega }$ in Eq. (23) still contains a parameter θ, and since parameter θ is estimated by $\hat{\theta }$ in Eq. (25), and considering ${\hat{\delta }}_{\Omega }={\left(X^{\prime }X\right)}^{-1}X^{\prime }y$. Therefore, the parameter $\delta_{\omega }$, which is estimated by ${\hat{\delta }}_{\omega }$ in Eq. (23) can be written as follows.

(26) $$\begin{array}{ccc} {\hat{\delta }}_{\omega } & = & {\left(X^{\prime }X\right)}^{-1}\left(X^{\prime }y-{a^{\prime }}_{l}{\left(a_{l}{\left(X^{\prime }X\right)}^{-1}{a^{\prime }}_{l}\right)}^{-1}a_{l}{\left(X^{\prime }X\right)}^{-1}X^{\prime }y\right)\\ & = & {\hat{\delta }}_{\Omega }-{\left(X^{\prime }X\right)}^{-1}{a^{\prime }}_{l}{\left(a_{l}{\left(X^{\prime }X\right)}^{-1}{a^{\prime }}_{l}\right)}^{-1}a_{l}{\hat{\delta }}_{\Omega } \end{array}$$

For the estimation of parameter $\sigma_{\omega }^{2}$, we can directly use the Maximum Likelihood Estimation (MLE) method, by solving $\frac{\partial \ln L(\omega )}{\partial \sigma_{\omega }^{2}}=0$. Based on the likelihood function under the parameter space ω in Eq. (17), we obtain lnL(ω) as follows.

(27) $$\ln L\left(\omega \right)=-\frac{n}{2}\ln\left(2\pi \sigma_{\omega }^{2}\right)-\frac{1}{2\sigma_{\omega }^{2}}{\left(y-X\delta_{\omega }\right)}^{\prime }\left(y-X\delta_{\omega }\right).$$

Therefore, based on Eq. (27), we obtain:

(28) $$\frac{\partial \ln L\left(\omega \right)}{\partial \sigma_{\omega }^{2}}=-\frac{n}{2\sigma_{\omega }^{2}}+\frac{1}{2\sigma_{\omega }^{4}}{\left(y-X\delta_{\omega }\right)}^{\prime }\left(y-X\delta_{\omega }\right)=0.$$

Furthermore, by elaborating on Eq. (28), and considering that $\delta_{\omega }$ is estimated by ${\hat{\delta }}_{\omega }$ in Eq. (26), the estimation of $\sigma_{\omega }^{2}$ is obtained as follows.

(29) $${\hat{\sigma }}_{\omega }^{2}=\frac{1}{n}{\left(y-X{\hat{\delta }}_{\omega }\right)}^{\prime }\left(y-X{\hat{\delta }}_{\omega }\right).$$

Therefore, based on (26), (29), we obtain the maximum likelihood under the parameter space ω as follows.

(30) $$\begin{array}{ccc} L\left(\hat{\omega }\right) & = & {\left(2\pi {\hat{\sigma }}_{\omega }^{2}\right)}^{-\frac{n}{2}}\exp\left(-\frac{1}{2{\hat{\sigma }}_{\omega }^{2}}{\left(y-X{\hat{\delta }}_{\omega }\right)}^{\prime }\left(y-X{\hat{\delta }}_{\omega }\right)\right)\\ & = & {\left(2\pi {\hat{\sigma }}_{\omega }^{2}\right)}^{-\frac{n}{2}}\exp\left(-\frac{{\left(y-X{\hat{\delta }}_{\omega }\right)}^{\prime }\left(y-X{\hat{\delta }}_{\omega }\right)}{2\frac{{\left(y-X{\hat{\delta }}_{\omega }\right)}^{\prime }\left(y-X{\hat{\delta }}_{\omega }\right)}{n}}\right)\\ & = & {\left(2\pi {\hat{\sigma }}_{\omega }^{2}\right)}^{-\frac{n}{2}}\exp\left(-\frac{n}{2}\right), \end{array}$$

where $L(\hat{\omega })$ in Eq. (30) is the maximum likelihood under the parameter space ω. Furthermore, based on the maximum likelihood under the parameter space ω in Eq. (30) and the maximum likelihood under the parameter space Ω in Eq. (20), we can express the likelihood ratio in Eq. (19) as follows.

(31) $$w\left(x_{i1},x_{i2},...,x_{ip};y_{i}\right)=\frac{L\left(\hat{\omega }\right)}{L\left(\hat{\Omega }\right)}=\frac{{\left(2\pi {\hat{\sigma }}_{\omega }^{2}\right)}^{-\frac{n}{2}}\exp\left(-\frac{n}{2}\right)}{{\left(2\pi {\hat{\sigma }}_{\Omega }^{2}\right)}^{-\frac{n}{2}}\exp\left(-\frac{n}{2}\right)}=\frac{{\left({\hat{\sigma }}_{\omega }^{2}\right)}^{-\frac{n}{2}}}{{\left({\hat{\sigma }}_{\Omega }^{2}\right)}^{-\frac{n}{2}}}.$$

Given that ${\hat{\sigma }}_{\omega }^{2}$ is provided in Eq. (29) and ${\hat{\sigma }}_{\Omega }^{2}$ is provided in Eq. (20), Eq. (31) can be written as follows.

(32) $$w\left(x_{i1},x_{i2},...,x_{ip};y_{i}\right)={\left(\frac{\frac{{\left(y-X{\hat{\delta }}_{\omega }\right)}^{\prime }\left(y-X{\hat{\delta }}_{\omega }\right)}{n}}{\frac{{\left(y-X{\hat{\delta }}_{\Omega }\right)}^{\prime }\left(y-X{\hat{\delta }}_{\Omega }\right)}{n}}\right)}^{-\frac{n}{2}}={\left(\frac{M\left({\hat{\delta }}_{\omega }\right)}{{\left(y-X{\hat{\delta }}_{\Omega }\right)}^{\prime }\left(y-X{\hat{\delta }}_{\Omega }\right)}\right)}^{-\frac{n}{2}},$$

where $M\left({\hat{\delta }}_{\omega }\right)={\left(y-X{\hat{\delta }}_{\omega }\right)}^{\prime }\left(y-X{\hat{\delta }}_{\omega }\right)=y^{\prime }y-2{{\hat{\delta }}^{\prime }}_{\omega }X^{\prime }y+{{\hat{\delta }}^{\prime }}_{\omega }X^{\prime }X{\hat{\delta }}_{\omega }$. Furthermore, since ${\hat{\delta }}_{\omega }$ is the estimated parameter under the parameter space of ω, as given in Eq. (26), $M({\hat{\delta }}_{\omega })$ can be written as follows.

$$\begin{array}{ccc} M\left({\hat{\delta }}_{\omega }\right) & = & y^{\prime }y-2{\left({\hat{\delta }}_{\Omega }-{\left(X^{\prime }X\right)}^{-1}{a^{\prime }}_{l}{\left(a_{l}{\left(X^{\prime }X\right)}^{-1}{a^{\prime }}_{l}\right)}^{-1}a_{l}{\hat{\delta }}_{\Omega }\right)}^{\prime }X^{\prime }y\\ & & +\;{\left({\hat{\delta }}_{\Omega }-{\left(X^{\prime }X\right)}^{-1}{a^{\prime }}_{l}{\left(a_{l}{\left(X^{\prime }X\right)}^{-1}{a^{\prime }}_{l}\right)}^{-1}a_{l}{\hat{\delta }}_{\Omega }\right)}^{\prime }X^{\prime }X\left({\hat{\delta }}_{\Omega }-{\left(X^{\prime }X\right)}^{-1}{a^{\prime }}_{l}{\left(a_{l}{\left(X^{\prime }X\right)}^{-1}{a^{\prime }}_{l}\right)}^{-1}a_{l}{\hat{\delta }}_{\Omega }\right)\\ & = & y^{\prime }y-2{{\hat{\delta }}^{\prime }}_{\Omega }X^{\prime }y+2{{\hat{\delta }}^{\prime }}_{\Omega }{a^{\prime }}_{l}{\left(a_{l}{\left(X^{\prime }X\right)}^{-1}{a^{\prime }}_{l}\right)}^{-1}a_{l}{\left(X^{\prime }X\right)}^{-1}X^{\prime }y\\ & & +\;{{\hat{\delta }}^{\prime }}_{\Omega }X^{\prime }X{\hat{\delta }}_{\Omega }-{{\hat{\delta }}^{\prime }}_{\Omega }X^{\prime }X{\left(X^{\prime }X\right)}^{-1}{a^{\prime }}_{l}{\left(a_{l}{\left(X^{\prime }X\right)}^{-1}{a^{\prime }}_{l}\right)}^{-1}a_{l}{\hat{\delta }}_{\Omega }-{{\hat{\delta }}^{\prime }}_{\Omega }{a^{\prime }}_{l}{\left(a_{l}{\left(X^{\prime }X\right)}^{-1}{a^{\prime }}_{l}\right)}^{-1}a_{l}{\left(X^{\prime }X\right)}^{-1}X^{\prime }X{\hat{\delta }}_{\Omega }\\ & & +\;{{\hat{\delta }}^{\prime }}_{\Omega }{a^{\prime }}_{l}{\left(a_{l}{\left(X^{\prime }X\right)}^{-1}{a^{\prime }}_{l}\right)}^{-1}a_{l}{\left(X^{\prime }X\right)}^{-1}X^{\prime }X{\left(X^{\prime }X\right)}^{-1}{a^{\prime }}_{l}{\left(a_{l}{\left(X^{\prime }X\right)}^{-1}{a^{\prime }}_{l}\right)}^{-1}a_{l}{\hat{\delta }}_{\Omega }\\ & = & y^{\prime }y-2{{\hat{\delta }}^{\prime }}_{\Omega }X^{\prime }y+3{{\hat{\delta }}^{\prime }}_{\Omega }{a^{\prime }}_{l}{\left(a_{l}{\left(X^{\prime }X\right)}^{-1}{a^{\prime }}_{l}\right)}^{-1}a_{l}{\hat{\delta }}_{\Omega }+{{\hat{\delta }}^{\prime }}_{\Omega }X^{\prime }X{\hat{\delta }}_{\Omega }-2{{\hat{\delta }}^{\prime }}_{\Omega }{a^{\prime }}_{l}{\left(a_{l}{\left(X^{\prime }X\right)}^{-1}{a^{\prime }}_{l}\right)}^{-1}a_{l}{\hat{\delta }}_{\Omega }\\ & = & y^{\prime }y-2{{\hat{\delta }}^{\prime }}_{\Omega }X^{\prime }y+{{\hat{\delta }}^{\prime }}_{\Omega }X^{\prime }X{\hat{\delta }}_{\Omega }+{{\hat{\delta }}^{\prime }}_{\Omega }{a^{\prime }}_{l}{\left(a_{l}{\left(X^{\prime }X\right)}^{-1}{a^{\prime }}_{l}\right)}^{-1}a_{l}{\hat{\delta }}_{\Omega }\\ & = & {\left(y-X{\hat{\delta }}_{\Omega }\right)}^{\prime }\left(y-X{\hat{\delta }}_{\Omega }\right)+{\left(a_{l}{\hat{\delta }}_{\Omega }\right)}^{\prime }{\left(a_{l}{\left(X^{\prime }X\right)}^{-1}{a^{\prime }}_{l}\right)}^{-1}\left(a_{l}{\hat{\delta }}_{\Omega }\right) \end{array}$$

Therefore, by substituting $M({\hat{\delta }}_{\omega })$ into Eq. (32), we obtain the likelihood ratio as follows.

(33) $$\begin{array}{ccc} w\left(x_{i1},x_{i2},...,x_{ip};y_{i}\right) & = & {\left(\frac{{\left(y-X{\hat{\delta }}_{\Omega }\right)}^{\prime }\left(y-X{\hat{\delta }}_{\Omega }\right)+{\left(a_{l}{\hat{\delta }}_{\Omega }\right)}^{\prime }{\left(a_{l}{\left(X^{\prime }X\right)}^{-1}{a^{\prime }}_{l}\right)}^{-1}\left(a_{l}{\hat{\delta }}_{\Omega }\right)}{{\left(y-X{\hat{\delta }}_{\Omega }\right)}^{\prime }\left(y-X{\hat{\delta }}_{\Omega }\right)}\right)}^{-\frac{n}{2}}\\ & = & {\left(1+\frac{{\left(a_{l}{\hat{\delta }}_{\Omega }\right)}^{\prime }{\left(a_{l}{\left(X^{\prime }X\right)}^{-1}{a^{\prime }}_{l}\right)}^{-1}\left(a_{l}{\hat{\delta }}_{\Omega }\right)}{{\left(y-X{\hat{\delta }}_{\Omega }\right)}^{\prime }\left(y-X{\hat{\delta }}_{\Omega }\right)}\right)}^{-\frac{n}{2}} \end{array}$$

Based on the likelihood ratio in Eq. (33), the statistical test for partial hypothesis testing in nonparametric regression model with approximation of a Fourier series function with the hypothesis form given in Eq. (11) is presented in Theorem 1.

Theorem 1 *Given the hypothesis form in*Eq. (11)*, by using the LRT method, the statistical test for testing*$H_{0}:a_{l}\delta =0$*against*$H_{1}:a_{l}\delta \neq 0$*is as follows.*

(34) $$Z=\frac{a_{l}\hat{\delta }}{\sqrt{\frac{Q}{r}\left(a_{l}{\left(X^{\prime }X\right)}^{-1}{a^{\prime }}_{l}\right)}}$$

where $Q={\left(y-X\hat{\delta }\right)}^{\prime }\left(y-X\hat{\delta }\right)$ and r is obtained in Theorem 2.

*Proof.* Noted that the partial hypothesis testing in nonparametric regression model with approximation of a Fourier series function employs the LRT method. According to Casella and Berger, the LRT method specifies a rejection region, denoted as $\left\{x_{i1},x_{i2},...,x_{ip};y_{i}|w\left(x_{i1},x_{i2},...,x_{ip};y_{i}\right)\leq a\right\}$, where a is a constant satisfying 0≤a≤1 [30]. Therefore, based on the rejection region of the LRT method, the likelihood ratio in Eq. (33) can be expressed as:

(35) $$\begin{array}{ccc} & & {\left(1+\frac{{\left(a_{l}{\hat{\delta }}_{\Omega }\right)}^{\prime }{\left(a_{l}{\left(X^{\prime }X\right)}^{-1}{a^{\prime }}_{l}\right)}^{-1}\left(a_{l}{\hat{\delta }}_{\Omega }\right)}{{\left(y-X{\hat{\delta }}_{\Omega }\right)}^{\prime }\left(y-X{\hat{\delta }}_{\Omega }\right)}\right)}^{-\frac{n}{2}}\leq a\\ & & \;\frac{{\left(a_{l}{\hat{\delta }}_{\Omega }\right)}^{\prime }{\left(a_{l}{\left(X^{\prime }X\right)}^{-1}{a^{\prime }}_{l}\right)}^{-1}\left(a_{l}{\hat{\delta }}_{\Omega }\right)}{{\left(y-X{\hat{\delta }}_{\Omega }\right)}^{\prime }\left(y-X{\hat{\delta }}_{\Omega }\right)}\geq {\left(a\right)}^{-\frac{2}{n}}-1 \end{array}$$

By multiplying Eq. (35) by r, where r represents the degrees of freedom as obtained later in Theorem 2, Eq. (35) can be written as:

(36) $$\frac{{\left(a_{l}{\hat{\delta }}_{\Omega }\right)}^{\prime }{\left(a_{l}{\left(X^{\prime }X\right)}^{-1}{a^{\prime }}_{l}\right)}^{-1}\left(a_{l}{\hat{\delta }}_{\Omega }\right)}{\frac{{\left(y-X{\hat{\delta }}_{\Omega }\right)}^{\prime }\left(y-X{\hat{\delta }}_{\Omega }\right)}{r}}\geq \left({\left(a\right)}^{-\frac{2}{n}}-1\right)r.$$

Since $a_{l}$ is a row vector and ${\hat{\delta }}_{\Omega }$ is a column vector, the product of $a_{l}{\hat{\delta }}_{\Omega }$ dan $a_{l}{\left(X^{\prime }X\right)}^{-1}{a^{\prime }}_{l}$ yield constants. Therefore, Eq. (36) can be written as:

(37) $$\frac{{\left(a_{l}{\hat{\delta }}_{\Omega }\right)}^{2}}{\frac{{\left(y-X{\hat{\delta }}_{\Omega }\right)}^{\prime }\left(y-X{\hat{\delta }}_{\Omega }\right)}{r}\left(a_{l}{\left(X^{\prime }X\right)}^{-1}{a^{\prime }}_{l}\right)}\geq \left({\left(a\right)}^{-\frac{2}{n}}-1\right)r.$$

Furthermore, Eq. (37) is squared on both sides, and since ${\hat{\delta }}_{\Omega }={\left(X^{\prime }X\right)}^{-1}X^{\prime }y$ equals $\hat{\delta }$ as of Eq. (6). Therefore, Eq. (37) can be written as:

(38) $$\begin{array}{c} \left|\frac{a_{l}\hat{\delta }}{\sqrt{\frac{{\left(y-X\hat{\delta }\right)}^{\prime }\left(y-X\hat{\delta }\right)}{r}\left(a_{l}{\left(X^{\prime }X\right)}^{-1}{a^{\prime }}_{l}\right)}}\right|\geq \sqrt{\left({\left(a\right)}^{-\frac{2}{n}}-1\right)r}\\ \left|Z\right|\geq a^{*} \end{array}$$

where $Z=\frac{a_{l}\hat{\delta }}{\sqrt{\frac{Q}{r}\left(a_{l}{\left(X^{\prime }X\right)}^{-1}{a^{\prime }}_{l}\right)}}$ with $Q={\left(y-X\hat{\delta }\right)}^{\prime }\left(y-X\hat{\delta }\right)$ and $a^{*}=\sqrt{({\left(a\right)}^{-\frac{2}{n}}-1)r}$. Based on Eq. (38), the rejection region for testing the hypothesis $H_{0}:a_{l}\delta =0$ against $H_{1}:a_{l}\delta \neq 0$ is $\left\{x_{i1},x_{i2},...,x_{ip};y_{i}∥Z|\leq a^{*}\right\}$. Therefore, the statistical test for testing $H_{0}:a_{l}\delta =0$ against $H_{1}:a_{l}\delta \neq 0$ can be defined as follows.

$$Z=\frac{a_{l}\hat{\delta }}{\sqrt{\frac{Q}{r}\left(a_{l}{\left(X^{\prime }X\right)}^{-1}{a^{\prime }}_{l}\right)}}$$

where Z is the statistical test for partial hypothesis testing with the hypothesis form given in Eq. (11).

#### Distribution of the statistical test

Based on Theorem 1, Z is the statistical test for partial hypothesis testing in nonparametric regression model with approximation of a Fourier series function using the hypothesis form in Eq. (11), where the rejection region for testing $H_{0}:a_{l}\delta =0$ against $H_{1}:a_{l}\delta \neq 0$ is $\left\{x_{i1},x_{i2},...,x_{ip};y_{i}∥Z|\leq a^{*}\right\}$, where $a^{*}$ contains a constant a. To determine the value of a we derive the distribution of the statistical test Z. The distribution of the statistical test Z is obtained based on Theorem 2. Before presenting Theorem 2, Lemma 1 is provided to facilitate the proof of Theorem 2.

Lemma 1 *Let*Z*is the statistical test given in*Theorem 1*then*$Z^{2}$*can be written in quadratic form as follows*.

$$Z^{2}=\frac{y^{\prime }\mathrm{My}}{\frac{y^{\prime }\left(I-U\right)y}{r}}$$

where $M=X{\left(X^{\prime }X\right)}^{-1}{a^{\prime }}_{l}{\left(a_{l}{\left(X^{\prime }X\right)}^{-1}{a^{\prime }}_{l}\right)}^{-1}a_{l}{\left(X^{\prime }X\right)}^{-1}X^{\prime }$ and U is given in Eq. (7).

*Proof.* Noted that Z is the statistical test obtained based on Theorem 1. Furthermore, if the statistical test Z in Eq. (34) is squared, it can be written as follows.

(39) $$Z^{2}={\left(\frac{a_{l}\hat{\delta }}{\sqrt{\frac{Q}{r}\left(a_{l}{\left(X^{\prime }X\right)}^{-1}{a^{\prime }}_{l}\right)}}\right)}^{2}=\frac{{\left(a_{l}\hat{\delta }\right)}^{2}}{\frac{{\left(y-X\hat{\delta }\right)}^{\prime }\left(y-X\hat{\delta }\right)}{r}\left(a_{l}{\left(X^{\prime }X\right)}^{-1}{a^{\prime }}_{l}\right)}=\frac{{\left(a_{l}\hat{\delta }\right)}^{\prime }{\left(a_{l}{\left(X^{\prime }X\right)}^{-1}{a^{\prime }}_{l}\right)}^{-1}\left(a_{l}\hat{\delta }\right)}{\frac{y^{\prime }y-2{\hat{\delta }}^{\prime }X^{\prime }y+{\hat{\delta }}^{\prime }X^{\prime }X\hat{\delta }}{r}}.$$

Since $\hat{\delta }={\left(X^{\prime }X\right)}^{-1}X^{\prime }y$, Eq. (39) can be simplified in quadratic form as follows.

$$Z^{2}=\frac{y^{\prime }X{\left(X^{\prime }X\right)}^{-1}{a^{\prime }}_{l}{\left(a_{l}{\left(X^{\prime }X\right)}^{-1}{a^{\prime }}_{l}\right)}^{-1}a_{l}{\left(X^{\prime }X\right)}^{-1}X^{\prime }y}{\frac{y^{\prime }y-y^{\prime }X{\left(X^{\prime }X\right)}^{-1}X^{\prime }y}{r}}=\frac{y^{\prime }\mathrm{My}}{\frac{y^{\prime }\left(I-U\right)y}{r}},$$

where $Z^{2}$ describes the quadratic form of Z with $y^{\prime }\mathrm{My}$ and $y^{\prime }\left(I-U\right)y$ are being quadratic in y.

Theorem 2 *Let*Z*denote the statistical test for testing*$H_{0}:a_{l}\delta =0$*against*$H_{1}:a_{l}\delta \neq 0$*. If the statistic*$Z^{2}$*follows an*F*distribution with degrees of freedom 1 and*r*, then the statistical test*Z*follows the*t*student distribution with*r*degrees of freedom as follows.*

$$Z=\frac{a_{l}{\hat{\delta }}_{\Omega }}{\sqrt{\frac{Q}{r}\left(a_{l}{\left(X^{\prime }X\right)}^{-1}{a^{\prime }}_{l}\right)}}∼t_{\left(r\right)}$$

where r=n−p(T+1)+1.

*Proof.* Noted that Z is the statistical test for testing $H_{0}:a_{l}\delta =0$ against $H_{1}:a_{l}\delta \neq 0$, where Z is given in Theorem 1. Furthermore, based on Lemma 1 we have the statistic $Z^{2}$ as follows.

$$Z^{2}={\left(\frac{a_{l}{\hat{\delta }}_{\Omega }}{\sqrt{\frac{Q}{r}\left(a_{l}{\left(X^{\prime }X\right)}^{-1}{a^{\prime }}_{l}\right)}}\right)}^{2}=\frac{y^{\prime }\mathrm{My}}{\frac{y^{\prime }\left(I-U\right)y}{r}}.$$

Let multiply the statistic $Z^{2}$ with $\frac{\sigma^{2}}{\sigma^{2}}$. Therefore, we have:

$$Z^{2}=\frac{\frac{y^{\prime }\mathrm{My}}{\sigma^{2}}}{\frac{y^{\prime }\left(I-U\right)y}{\sigma^{2}r}}.$$

The statistic $Z^{2}$ follows an F distribution with degrees of freedom 1 and r if and only if $\frac{y^{\prime }\mathrm{My}}{\sigma^{2}}$ follows the chi-square distribution with 1 degree of freedom and $\frac{y^{\prime }\left(I-U\right)y}{\sigma^{2}}$ follows the chi-square distribution with r degrees of freedom, where $y^{\prime }\mathrm{My}$ and $y^{\prime }\left(I-U\right)y$ are independent. Furthermore, since $y^{\prime }\mathrm{My}$ is being quadratic in y then to prove that $\frac{y^{\prime }\mathrm{My}}{\sigma^{2}}$ follows the chi-square distribution with 1 degree of freedom is the same way as showing that matrix M is symmetry and idempotent with trace(M)=1.

For matrix M is symmetry then the product of $M^{\prime }=M$ as follows.

(40) $$\begin{array}{ccc} M^{\prime } & = & {\left(X{\left(X^{\prime }X\right)}^{-1}{a^{\prime }}_{l}{\left(a_{l}{\left(X^{\prime }X\right)}^{-1}{a^{\prime }}_{l}\right)}^{-1}a_{l}{\left(X^{\prime }X\right)}^{-1}X^{\prime }\right)}^{\prime }\\ & = & X{\left(X^{\prime }X\right)}^{-1}{a^{\prime }}_{l}{\left(a_{l}{\left(X^{\prime }X\right)}^{-1}{a^{\prime }}_{l}\right)}^{-1}a_{l}{\left(X^{\prime }X\right)}^{-1}X^{\prime }=M \end{array}$$

For matrix M is idempotent then the product of $M^{2}=M$ as follows.

(41) $$\begin{array}{ccc} M^{2} & = & M^{\prime }\times M=M\times M\\ & = & X{\left(X^{\prime }X\right)}^{-1}{a^{\prime }}_{l}{\left(a_{l}{\left(X^{\prime }X\right)}^{-1}{a^{\prime }}_{l}\right)}^{-1}a_{l}{\left(X^{\prime }X\right)}^{-1}X^{\prime }X{\left(X^{\prime }X\right)}^{-1}{a^{\prime }}_{l}{\left(a_{l}{\left(X^{\prime }X\right)}^{-1}{a^{\prime }}_{l}\right)}^{-1}a_{l}{\left(X^{\prime }X\right)}^{-1}X^{\prime }\\ & = & X{\left(X^{\prime }X\right)}^{-1}{a^{\prime }}_{l}{\left(a_{l}{\left(X^{\prime }X\right)}^{-1}{a^{\prime }}_{l}\right)}^{-1}a_{l}{\left(X^{\prime }X\right)}^{-1}X^{\prime }=M \end{array}$$

For trace(M)=1, let $U_{1}=X{\left(X^{\prime }X\right)}^{-1}a^{\prime }$ and $U_{2}=a_{l}{\left(X^{\prime }X\right)}^{-1}X^{\prime }$, since $a_{l}{\left(X^{\prime }X\right)}^{-1}{a^{\prime }}_{l}$ is a constant then trace(M) is obtained as follows.

$$\begin{array}{c} trace\left(M\right)=trace\left(U_{1}{\left(a_{l}{\left(X^{\prime }X\right)}^{-1}{a^{\prime }}_{l}\right)}^{-1}U_{2}\right)=trace\left(\frac{U_{1}U_{2}}{a_{l}{\left(X^{\prime }X\right)}^{-1}{a^{\prime }}_{l}}\right)=trace\left(\frac{U_{2}U_{1}}{a_{l}{\left(X^{\prime }X\right)}^{-1}{a^{\prime }}_{l}}\right)\\ \;\;\;\;\;\;\;\;\;\;\;\;\;\;\;\;\;\;\;\;\;\;\;\;\;\;=trace\left(\frac{a_{l}{\left(X^{\prime }X\right)}^{-1}a^{\prime }}{a_{l}{\left(X^{\prime }X\right)}^{-1}{a^{\prime }}_{l}}\right)=trace\left(1\right)=1 \end{array}$$

Based on (40), (41), matrix M is symmetry and idempotent with trace(M)=1. Therefore, $\frac{y^{\prime }\mathrm{My}}{\sigma^{2}}$ follows the chi-square distribution with 1 degree of freedom as follows.

(42) $$\frac{y^{\prime }\mathrm{My}}{\sigma^{2}}∼\chi_{\left(1\right)}^{2}.$$

According to Ramli, et al., $\frac{y^{\prime }\left(I-U\right)y}{\sigma^{2}}$ follows the chi-square distribution with r degrees of freedom as follows [19].

(43) $$\frac{y^{\prime }\left(I-N\right)y}{\sigma^{2}}∼\chi_{\left(r\right)}^{2},$$

where r=n−p(T+1)+1. Furthermore, for $y^{\prime }\mathrm{My}$ and $y^{\prime }\left(I-U\right)y$ are independent. This can be proved by showing the product of matrices M×(I−U)=0 as follows.

$$\begin{array}{c} M\times \left(I-U\right)=M-M\times U=X{\left(X^{\prime }X\right)}^{-1}{a^{\prime }}_{l}{\left(a_{l}{\left(X^{\prime }X\right)}^{-1}{a^{\prime }}_{l}\right)}^{-1}a_{l}{\left(X^{\prime }X\right)}^{-1}X^{\prime }\\ \;\;\;\;\;\;\;\;\;\;\;\;\;\;\;\;\;\;\;\;\;\;\;\;\;\;\;\;\;\;\;\;\;\;\;-X{\left(X^{\prime }X\right)}^{-1}{a^{\prime }}_{l}{\left(a_{l}{\left(X^{\prime }X\right)}^{-1}{a^{\prime }}_{l}\right)}^{-1}a_{l}{\left(X^{\prime }X\right)}^{-1}X^{\prime }X{\left(X^{\prime }X\right)}^{-1}X^{\prime }\\ \;\;\;\;\;\;\;\;\;\;\;\;\;\;\;\;\;\;\;\;\;\;\;\;\;\;\;\;\;\;=X{\left(X^{\prime }X\right)}^{-1}{a^{\prime }}_{l}{\left(a_{l}{\left(X^{\prime }X\right)}^{-1}{a^{\prime }}_{l}\right)}^{-1}a_{l}{\left(X^{\prime }X\right)}^{-1}X^{\prime }-X{\left(X^{\prime }X\right)}^{-1}{a^{\prime }}_{l}{\left(a_{l}{\left(X^{\prime }X\right)}^{-1}{a^{\prime }}_{l}\right)}^{-1}a_{l}{\left(X^{\prime }X\right)}^{-1}X^{\prime }\\ \;\;\;\;\;\;\;\;\;\;\;\;\;\;\;\;\;\;\;\;\;\;\;\;\;\;\;\;\;\;\;=0 \end{array}$$

Since the product of matrices M and (I−U) equals 0, it implies that $y^{\prime }\mathrm{My}$ and $y^{\prime }\left(I-U\right)y$ are independent. Based on (42), (43), since $\frac{y^{\prime }\mathrm{My}}{\sigma^{2}}∼\chi_{\left(1\right)}^{2}$ and $\frac{y^{\prime }\left(I-U\right)y}{\sigma^{2}}∼\chi_{\left(r\right)}^{2}$, where $y^{\prime }\mathrm{My}$ and $y^{\prime }\left(I-U\right)y$ are independent. Therefore, we obtain the statistic $Z^{2}$ follows an F distribution with degrees of freedom 1 and r as follows.

$$Z^{2}=\frac{\frac{y^{\prime }\mathrm{My}}{\sigma^{2}}}{\frac{y^{\prime }\left(I-U\right)y}{\sigma^{2}r}}=\frac{y^{\prime }\mathrm{My}}{\frac{y^{\prime }\left(I-U\right)y}{r}}∼F_{\left(1,r\right)}.$$

Since $Z^{2}∼F_{\left(1,r\right)}$ and based on Lemma 1, where $\sqrt{Z^{2}}=Z$. Therefore, the statistical test Z follows the t student distribution with r degrees of freedom as follows.

$$\sqrt{Z^{2}}=\sqrt{\frac{y^{\prime }\mathrm{My}}{\frac{y^{\prime }\left(I-U\right)y}{r}}}=\sqrt{{\left(\frac{a_{l}\hat{\delta }}{\sqrt{\frac{Q}{r}\left(a_{l}{\left(X^{\prime }X\right)}^{-1}{a^{\prime }}_{l}\right)}}\right)}^{2}}=\frac{a_{l}\hat{\delta }}{\sqrt{\frac{Q}{r}\left(a_{l}{\left(X^{\prime }X\right)}^{-1}{a^{\prime }}_{l}\right)}}∼t_{\left(r\right)}.$$

#### The rejection region for the null hypothesis

The purpose of determining the rejection region for the null hypothesis is to find out whether the null hypothesis is rejected or fails to be rejected at a significance level α. Noted that partial hypothesis testing in nonparametric regression model with approximation of a Fourier series function, with the hypothesis form in Eq. (11), uses the LRT method. Since the LRT method has the rejection region of $\left\{x_{i1},x_{i2},...,x_{ip};y_{i}|w\left(x_{i1},x_{i2},...,x_{ip};y_{i}\right)\leq a\right\}$, where a is a constant. Therefore, based on Theorem 1, the critical region for testing $H_{0}:a_{l}\delta =0$ against $H_{1}:a_{l}\delta \neq 0$ can be written as follows.

(44) $$\begin{array}{c} C=\left\{x_{i1},x_{i2},...,x_{ip};y_{i}|w\left(x_{i1},x_{i2},...,x_{ip};y_{i}\right)\leq a\right\}\\ \;\;\;\;\;=\left\{x_{i1},x_{i2},...,x_{ip},y_{i}|\left|\frac{a_{l}\hat{\delta }}{\sqrt{\frac{Q}{r}\left(a_{l}{\left(X^{\prime }X\right)}^{-1}{a^{\prime }}_{l}\right)}}\right|\geq \sqrt{\left({\left(a\right)}^{-\frac{2}{n}}-1\right)r}\right\}\\ \;\;\;\;\;=\left\{x_{i1},x_{i2},...,x_{ip},y_{i}|\left|Z\right|\geq a^{*}\right\} \end{array}$$

If a significance level α is given, then based on Eq. (44), the critical region for rejecting the null hypothesis for testing $H_{0}:a_{l}\delta =0$ against $H_{1}:a_{l}\delta \neq 0$ is given by:

(45) $$\begin{array}{c} \alpha =P\left(\text{Reject}\;H_{0}|H_{0}\;\text{True}\right)\\ \alpha =P\left(\left|Z\right|\geq a^{*}|a_{l}\delta =0\right) \end{array}$$

Based on Theorem 2, we know that the statistical test Z follows a t student distribution with r degrees of freedom, where r=n−(p(T+1)+1). Furthermore, since $a^{*}=\left({\left(a\right)}^{-\frac{2}{n}}-1\right)r$, where a is a constant, then based on Eq. (45), $a^{*}$ can be obtained by integrating the probability density function of the t student distribution with r degrees of freedom at a significance level α as follows.

(46) $$\int_{a^{*}}^{\infty }f\left(t\right)dt=\int_{-\infty }^{a^{*}}f\left(t\right)dt=\frac{\alpha }{2},$$

where f(t) is the probability density function of the t student distribution with r degrees of freedom. Based on Eq. (46), $a^{*}$ represents a statistic value of the t student distribution with r degrees of freedom at the significance level $\frac{\alpha }{2}$, denoted as $a^{*}=t_{\left(\frac{\alpha }{2},r\right)}$. Therefore, based on Eq. (45), the critical region for rejecting the null hypothesis for testing $H_{0}:a_{l}\delta =0$ against $H_{1}:a_{l}\delta \neq 0$ can be written as Eq. (47).

(47) $$\alpha =P\left(\left|Z\right|\geq t_{\left(\frac{\alpha }{2},r\right)}|a_{l}\delta =0\right).$$

Based on Eq. (47), the null hypothesis is rejected at a significance level α if and only if $\left|Z\right|\geq t_{\left(\frac{\alpha }{2},r\right)}$ or the probability of $P(\left|Z\right|\geq t_{\left(\frac{\alpha }{2},r\right)})$ is less than α.

### Method validation

#### Real data application

Statistical inference on nonparametric regression model with approximation of a Fourier series function have been theoretically discussed in the previous section. To apply these concepts to real data, we gathered life expectancy data from all districts and cities in East Java Province. East Java has 29 districts and 9 cities, providing a total of n=38 observations for this research. The variables used in this research are divided into two categories: the response variable (y) and the predictor variables (x). The response variable is the life expectancy data of 38 districts and cities in East Java Province. The predictor variables used in the study include, poverty percentage $\left(x_{1}\right)$, open unemployment rate $\left(x_{2}\right)$, labor force participation rate $\left(x_{3}\right)$, average years of schooling $\left(x_{4}\right)$, and population percentage $\left(x_{5}\right)$. Life expectancy and predictor variables were sourced from the website of Badan Pusat Statistik (BPS) for East Java Province in 2022.

**Fig. 1:**
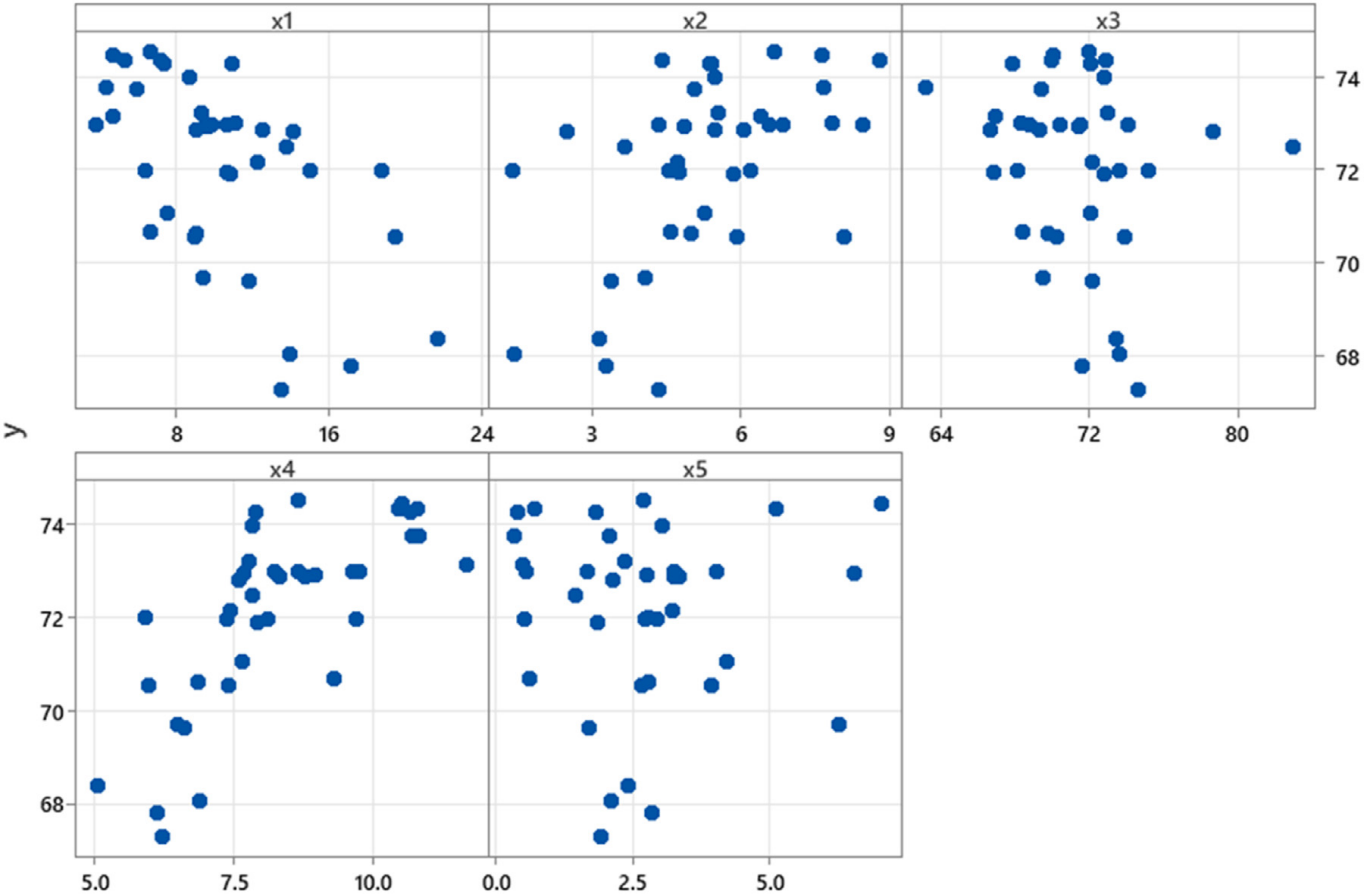
Scatter Plots between Response and Predictor Variables.

Before proceeding with modelling life expectancy data in East Java province, we first examined the relationship between life expectancy and each predictor variable. This preliminary investigation aims to discern the patterns exhibited by each predictor, thereby guiding the selection of an appropriate analytical approach. The relationship between life expectancy data and each predictor variable is expected to exhibit both unknown patterns and repeated patterns at certain intervals. Therefore, life expectancy data can be modelled using the nonparametric regression model with approximation of a Fourier series function. Furthermore, we visually explore the relationships between life expectancy and the predictor variables through scatter plots. The scatter plots illustrating the relationship between life expectancy data and variables poverty percentage $\left(x_{1}\right)$, open unemployment rate $\left(x_{2}\right)$, labor force participation rate $\left(x_{3}\right)$, average years of schooling $\left(x_{4}\right)$, and population percentage $\left(x_{5}\right)$ are presented in Fig. 1.

Based on Fig. 1, the relationship between life expectancy data and each predictor variable does not exhibit a specific pattern such as linear or nonlinear, which are typically assumed in parametric regression models. Therefore, the relationship pattern between life expectancy data and each predictor variable is considered to be unknown. Furthermore, to model life expectancy data with each predictor variable, a suitable approach is to use a nonparametric regression model. One effective estimator within nonparametric regression, particularly for curve estimation of life expectancy data is a Fourier series function. Based on Eq. (4) with n=38 dan p=5, the general form of the nonparametric regression model with approximation of a Fourier series function for life expectancy data in East Java Province during 2022 can be written as follows.

$$\begin{array}{cc} y_{i}=\sum_{j=1}^{5}\left(\frac{\mu_{j}}{2}+\beta_{j}x_{ij}+\sum_{t=1}^{T}\delta_{tj}\cos\left(tx_{ij}\right)\right)+\varepsilon_{i}, & i=1,2,...,38 \end{array}$$

Since the parameter estimates in nonparametric regression model with approximation of a Fourier series function depend on the number of oscillation parameters T, achieving optimal estimation results involves selecting the optimal T. The optimal T is determined using the GCV method, where the optimal T is identified based on the smallest GCV value. In this research, the maximum number of T is for T=5. Furthermore, the selection of T in this research is approached in two scenarios, where T is consistent across all predictor variables and T varies for each predictor variable. Based on the analysis results, the GCV values obtained when T is consistent across all predictor variables are presented in Table 2.

**Table 2:** The GCV values when T is consistent across all predictor variables.

Nilai T	GCV	MSE	MAE	RMSE	MAPE	$R^{2}$ (%)
1	1.992	1.006	0.830	1.003	0.012	73.27
2	1.641	0.550	0.609	0.742	0.008	85.38
3	1.748	0.350	0.445	0.592	0.006	90.70
4	1.902	0.190	0.337	0.436	0.005	94.96
5	3.181	0.108	0.265	0.329	0.004	97.13

Table 2 shows the values of GCV, $R^{2}$, Mean Square Error (MSE), Mean Absolute Error (MAE), Root Mean Square Error (RMSE), and Mean Absolute Percent Error (MAPE) for each T, where T is consistent across all predictor variables. According to Table 2, the smallest GCV value obtained is 1.641. Therefore, the optimal number of T is determined to be T=2 for all predictor variables. Furthermore, due to different behavioural changes observed in each predictor variable concerning the response variable, a second scenario was explored in this research where T varies for each predictor variable. By setting T=5 as the maximum, the combination of T for each predictor variable is selected when the maximum is T=2,T=3,T=4 and T=5. Based on the analysis results presented in Table 3, the minimum GCV values are obtained for each maximum T, specifically T=2,T=3,T=4 and T=5.

**Table 3:** The minimum GCV values for the optimal combination of T.

Maximum T	Optimal Combinations of T					Minimum GCV	MSE	MAE	RMSE	MAPE	$R^{2}$ (%)
	$x_{1}$	$x_{2}$	$x_{3}$	$x_{4}$	$x_{5}$						
2	T=2	T=2	T=2	T=2	T=1	1.557	0.571	0.622	0.755	0.009	84.84
3	T=2	T=2	T=3	T=3	T=2	1.345	0.373	0.466	0.611	0.007	90.10
4	T=1	T=2	T=4	T=3	T=4	1.025	0.230	0.391	0.480	0.005	93.89
5	T=1	T=2	T=4	T=3	T=5	0.706	0.141	0.320	0.376	0.004	96.24

Table 3 only shows the minimum GCV values as well as $R^{2}$, MSE, MAE, RMSE, and MAPE values for each optimal T combination. This is due to the substantial number of combinations for each maximum T, there are 32 combinations for T=2, 243 combinations for T=3, 1024 combinations for T=4, and 3125 combinations for T=5. For example, for a maximum of T=3, there are 243 combinations. From these 243 combinations, the smallest GCV value was 1.345, obtained with the combination T=2 for variable $x_{1},\;T=2$ for variable $x_{2},\;T=3$ for variable $x_{3},\;T=3$ for variable $x_{4}$, and T=2 for variable $x_{5}$. Based on Table 3, we obtain the smallest GCV value of 0.706, which corresponds to the maximum T=5 with the optimal combination of T being T=1 for variable $x_{1},\;T=2$ for variable $x_{2},\;T=4$ for variable $x_{3},\;T=3$ for variable $x_{4}$, and T=5 for variable $x_{5}$.

Overall, based on Table 2, Table 3, comparing the minimum GCV value when T is consistent across all predictor variables and when T varies for each predictor variable, the smallest GCV value is 0.706. This minimum GCV value is achieved with the combination T=1 for variable $x_{1},\;T=2$ for variable $x_{2},\;T=4$ for variable $x_{3},\;T=3$ for variable $x_{4}$, and T=5 for variable $x_{5}$. Therefore, we obtain the nonparametric regression model with approximation of a Fourier series function for life expectancy data in East Java Province during 2022 as follows.

(48) $$\begin{array}{c} y_{i}=\frac{\mu_{1}}{2}+\beta_{1}x_{i1}+\delta_{11}\cos\left(x_{i1}\right)+\frac{\mu_{2}}{2}+\beta_{2}x_{i2}+\sum_{t=1}^{2}\delta_{t2}\cos\left(tx_{i2}\right)+\frac{\mu_{3}}{2}+\beta_{3}x_{i3}+\sum_{t=1}^{4}\delta_{t3}\cos\left(tx_{i3}\right)+\frac{\mu_{4}}{2}+\beta_{4}x_{i4}\\ \;\;\;\;\;\;\;\;+\sum_{t=1}^{3}\delta_{t4}\cos\left(tx_{i4}\right)+\frac{\mu_{5}}{2}+\beta_{5}x_{i5}+\sum_{t=1}^{5}\delta_{t5}\cos\left(tx_{i5}\right)+\varepsilon_{i},\;i=1,2,...,38 \end{array}$$

Furthermore, the estimated parameters in nonparametric regression model with approximation of a Fourier series function for life expectancy data in East Java Province during 2022 are presented in Table 4.

**Table 4:** Parameter estimation.

Parameter	Estimation	Parameter	Estimation	Parameter	Estimation
$\mu_{1}$	20.350	$\delta_{13}$	−0.334	$\mu_{5}$	20.350
$\beta_{1}$	0.242	$\delta_{23}$	1.390	$\beta_{5}$	−0.171
$\delta_{11}$	−1.480	$\delta_{33}$	−0.656	$\delta_{15}$	0.991
$\mu_{2}$	20.350	$\delta_{43}$	0.902	$\delta_{25}$	−0.072
$\beta_{2}$	0.148	$\mu_{4}$	20.350	$\delta_{35}$	−1.425
$\delta_{12}$	1.438	$\beta_{4}$	1.014	$\delta_{45}$	−0.478
$\delta_{22}$	−0.554	$\delta_{14}$	−0.498	$\delta_{55}$	0.688
$\mu_{3}$	20.350	$\delta_{24}$	−0.200		
$\beta_{3}$	0.140	$\delta_{34}$	0.471		

Noted that life expectancy data in East Java Province is modelled using the nonparametric regression model with approximation of a Fourier series function as given in Eq. (48). Furthermore, to determine whether the parameters in Eq. (48) simultaneously influence the model, simultaneous hypothesis testing is conducted. Based on Eq. (48), the hypothesis form for simultaneous hypothesis testing in nonparametric regression model with approximation of a Fourier series function for life expectancy data in East Java Province can be expressed as follows.

$$H_{0}:\mu_{1}=...=\mu_{5}=\beta_{1}=...=\beta_{5}=\delta_{11}=\delta_{12}=\delta_{22}=\delta_{13}=...=\delta_{43}=\delta_{14}=...=\delta_{34}=\delta_{15}=...=\delta_{55}=0$$

$H_{1}:$ there is at least one parameter that is not equal to 0.

Since Λ is the statistical test for simultaneous hypothesis testing in nonparametric regression model with approximation of a Fourier series function, as specified in Eq. (10), where Λ follows an F distribution with degrees of freedom p(T+1)+1 and n−(p(T+1)+1). Therefore, based on analysis results for p=5 and n=38 with the optimal combination of T given in Table 3, we obtain the statistical test Λ value of 29,767.82 with a P−value of $7.384\times 10^{-35}$. Furthermore, using a significance level α of 5 %, we obtain an $F_{\left(0.05,21,17\right)}$ value of 2.219. Since the statistic value of Λ is greater than the statistic value of $F_{\left(0.05,21,17\right)}$ or alternatively, since the P−value is less than 5 %, we reject the null hypothesis. Therefore, it can be concluded that at a significance level of 5 %, there is evidence that at least one parameter in Eq. (48) is not equal to 0. In other words, the parameters in the nonparametric regression model with approximation of a Fourier series function for life expectancy data in East Java Province simultaneously have a significant influence.

Furthermore, following the simultaneous hypothesis testing which concluded that at least one parameter is significant in the model, the next step is to determine which specific parameters are significant through partial hypothesis testing. Based on the nonparametric regression model with approximation of a Fourier series function for life expectancy data in East Java Province as described in Eq. (48), the hypotheses form for partial hypothesis testing are provided in Table 5.

**Table 5:** Partial Hypotheses Form.

Null Hypothesis	Alternative Hypothesis	Null Hypothesis	Alternative Hypothesis	Null Hypothesis	Alternative Hypothesis
$H_{0}:\mu_{1}=0$	$H_{1}:\mu_{1}\neq 0$	$H_{0}:\beta_{5}=0$	$H_{1}:\beta_{5}\neq 0$	$H_{0}:\delta_{24}=0$	$H_{1}:\delta_{24}\neq 0$
$H_{0}:\mu_{2}=0$	$H_{1}:\mu_{2}\neq 0$	$H_{0}:\delta_{11}=0$	$H_{1}:\delta_{11}\neq 0$	$H_{0}:\delta_{34}=0$	$H_{1}:\delta_{34}\neq 0$
$H_{0}:\mu_{3}=0$	$H_{1}:\mu_{3}\neq 0$	$H_{0}:\delta_{12}=0$	$H_{1}:\delta_{12}\neq 0$	$H_{0}:\delta_{15}=0$	$H_{1}:\delta_{15}\neq 0$
$H_{0}:\mu_{4}=0$	$H_{1}:\mu_{4}\neq 0$	$H_{0}:\delta_{22}=0$	$H_{1}:\delta_{22}\neq 0$	$H_{0}:\delta_{25}=0$	$H_{1}:\delta_{25}\neq 0$
$H_{0}:\mu_{5}=0$	$H_{1}:\mu_{5}\neq 0$	$H_{0}:\delta_{13}=0$	$H_{1}:\delta_{13}\neq 0$	$H_{0}:\delta_{35}=0$	$H_{1}:\delta_{35}\neq 0$
$H_{0}:\beta_{1}=0$	$H_{1}:\beta_{1}\neq 0$	$H_{0}:\delta_{23}=0$	$H_{1}:\delta_{23}\neq 0$	$H_{0}:\delta_{45}=0$	$H_{1}:\delta_{45}\neq 0$
$H_{0}:\beta_{2}=0$	$H_{1}:\beta_{2}\neq 0$	$H_{0}:\delta_{33}=0$	$H_{1}:\delta_{33}\neq 0$	$H_{0}:\delta_{55}=0$	$H_{1}:\delta_{55}\neq 0$
$H_{0}:\beta_{3}=0$	$H_{1}:\beta_{3}\neq 0$	$H_{0}:\delta_{43}=0$	$H_{1}:\delta_{43}\neq 0$		
$H_{0}:\beta_{4}=0$	$H_{1}:\beta_{4}\neq 0$	$H_{0}:\delta_{14}=0$	$H_{1}:\delta_{14}\neq 0$		

Table 5 describes the hypothesis forms for partial hypothesis testing in nonparametric regression model with approximation of a Fourier series function for life expectancy data in East Java Province during 2022, where the general model is given in Eq. (48). Furthermore, according to Theorem 1, Z is the statistical test for partial hypothesis testing in nonparametric regression model with approximation of a Fourier series function, with Z defined by Eq. (34). Furthermore, based on Theorem 2, it is known that the statistical test Z follows a t student distribution with r degrees of freedom. Based on the analysis results for l=1,2,...25,p=5,n=38 and the optimal combination of T given in Table 3, we obtain the statistic values of |Z| and the P−value as presented in Table 6.

**Table 6:** Partial Hypothesis Testing.

Parameter	Estimation	Statistic |Z|	P-value	Decision
$\mu_{1}$	20.350	12.331	**0.000**	Reject $H_{0}$
$\beta_{1}$	0.242	3.581	**0.002**	Reject $H_{0}$
$\delta_{11}$	−1.480	6.067	**0.000**	Reject $H_{0}$
$\mu_{2}$	20.350	12.331	**0.000**	Reject $H_{0}$
$\beta_{2}$	0.148	1.945	0.069	Failed to Reject $H_{0}$
$\delta_{12}$	1.438	5.672	**0.000**	Reject $H_{0}$
$\delta_{22}$	−0.554	3.488	**0.003**	Reject $H_{0}$
$\mu_{3}$	20.350	12.331	**0.000**	Reject $H_{0}$
$\beta_{3}$	0.140	2.925	**0.009**	Reject $H_{0}$
$\delta_{13}$	−0.334	2.079	0.053	Failed to Reject $H_{0}$
$\delta_{23}$	1.390	6.212	**0.000**	Reject $H_{0}$
$\delta_{33}$	−0.656	3.554	**0.002**	Reject $H_{0}$
$\delta_{43}$	0.902	4.538	**0.000**	Reject $H_{0}$
$\mu_{4}$	20.350	12.331	**0.000**	Reject $H_{0}$
$\beta_{4}$	1.014	5.839	**0.000**	Reject $H_{0}$
$\delta_{14}$	−0.498	2.337	**0.032**	Reject $H_{0}$
$\delta_{24}$	−0.200	1.104	0.285	Failed to Reject $H_{0}$
$\delta_{34}$	0.471	2.780	**0.013**	Reject $H_{0}$
$\mu_{5}$	20.350	12.331	**0.000**	Reject $H_{0}$
$\beta_{5}$	−0.171	2.130	**0.048**	Reject $H_{0}$
$\delta_{15}$	0.991	3.653	**0.002**	Reject $H_{0}$
$\delta_{25}$	−0.072	0.279	0.783	Failed to Reject $H_{0}$
$\delta_{35}$	−1.425	4.593	**0.000**	Reject $H_{0}$
$\delta_{45}$	−0.478	2.399	**0.028**	Reject $H_{0}$
$\delta_{55}$	0.688	3.263	**0.005**	Reject $H_{0}$

Table 6 describes the partial hypothesis testing for all parameters in Eq. (48) and shows the estimation, the statistical test, the P−value, and the decision of each parameter. Furthermore, based on Eq. (47), the null hypothesis in partial hypothesis testing in nonparametric regression model with approximation of a Fourier series function is rejected at the significance level α if and only if the statistic value |Z| is greater than the statistic value of $t_{\left(\frac{\alpha }{2},r\right)}$, or alternatively, if the P−value is less than α. Furthermore, using a significance level α of 5 % and for r=17, we obtain the statistic value $t_{\left(0.025,17\right)}$ of 2.11. Based on Table 6, the decisions to reject the null hypothesis or fail to reject the null hypothesis are made by comparing each statistic value |Z| with the statistic value $t_{\left(0.025,17\right)}$ of 2.11 or alternatively, by comparing each P−value with α of 5 %. For example, for parameter $\beta_{1}$, we obtain the statistic value |Z| of 3.581. This value is greater than 2.11, or the P−value of 0.002 is <5 %. Therefore, the decision is to reject the null hypothesis, indicating that parameter $\beta_{1}$ has a significant influence on the model. Overall, at a significance level of 5 %, all parameters have a significant influence on the model, except for parameters $\beta_{2},\;\delta_{13},\;\delta_{24}$ and $\delta_{25}$, which do not have a statistically significant influence on the model. Although several parameters are not significant, overall, the predictor variables significantly influence the life expectancy data at a 5 % significance level.

#### Model interpretation

The life expectancy data was modelled using the nonparametric regression model with approximation of a Fourier series function, where theoretically the model depends on the optimal number of oscillation parameter T. In this research, selecting the optimal number of oscillation parameter T is based on the smallest GCV value. The smallest GCV value was obtained as 0.706, with the optimal combinations of T given in Table 3. Therefore, the nonparametric regression model with approximation of a Fourier series function for the life expectancy data is written in Eq. (48), where the parameters estimation provided in Table 4. Furthermore, based on Eq. (48) and Table 4, the model estimation for the life expectancy data is given as follows.

$$\begin{array}{c} {\hat{y}}_{i}=50.875+0.242x_{i1}-1.480\cos\left(x_{i1}\right)+0.148x_{i2}+1.438\cos\left(x_{i2}\right)-0.554\cos\left(2x_{i2}\right)+0.140x_{i3}-0.334\cos\left(x_{i3}\right)\\ \;\;\;\;\;\;\;\;\;\;\;+1.390\cos\left(2x_{i3}\right)-0.656\cos\left(3x_{i3}\right)+0.902\cos\left(4x_{i3}\right)+1.014x_{i4}-0.498\cos\left(x_{i4}\right)-0.200\cos\left(2x_{i4}\right)+0.471\cos\left(3x_{i4}\right)\\ \;\;\;\;\;\;\;\;\;\;\;-0.171x_{i5}+0.991\cos\left(x_{i5}\right)-0.072\cos\left(2x_{i5}\right)-1.425\cos\left(3x_{i5}\right)-0.478\cos\left(4x_{i5}\right)+0.688\cos\left(5x_{i5}\right) \end{array}$$

where ${\hat{y}}_{i}$ is a model estimation for the life expectancy data in East Java Province during 2022 with i=1,2,...,38. The model estimation produces an $R^{2}$ value of 96.24 %, MSE value of 0.141, and other values as MAE, RMSE, MAPE are given in Table 3. This indicates that 96.24 % of the predictor variables are able to explain the response variable or simultaneously the predictor variables have an effect of 96.24 % on the response variable, with a residual variance of 0.141, indicating the variability in the response variable that is not explained by the model. This is a strong indication that the model fits the data well. Moreover, the model estimation can also be interpreted graphically by plotting the actual values of the response variable against the predicted values.

**Fig. 2:**
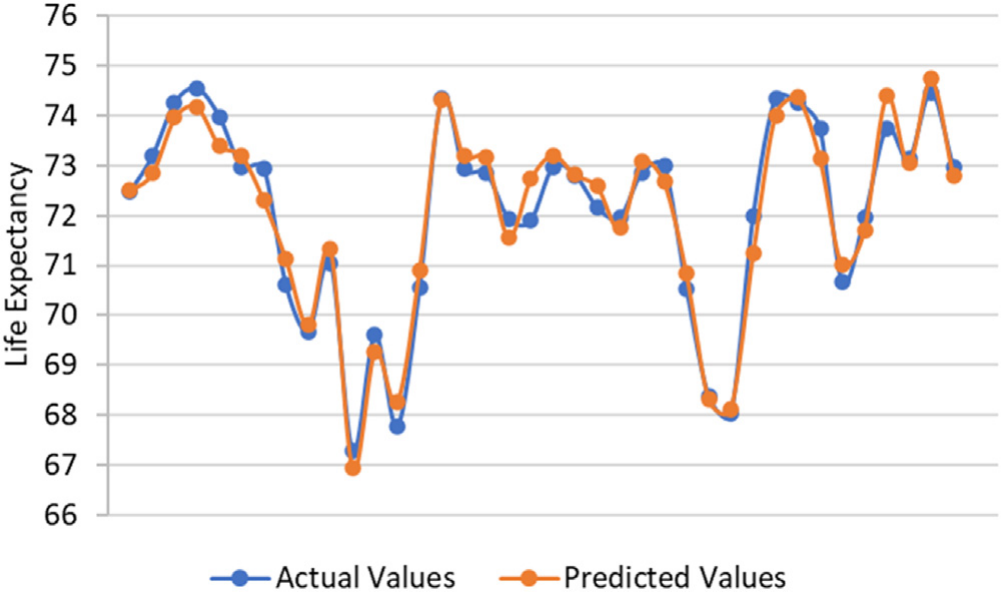
Comparison between Actual and Predicted Values of the Response Variable.

**Fig. 3:**
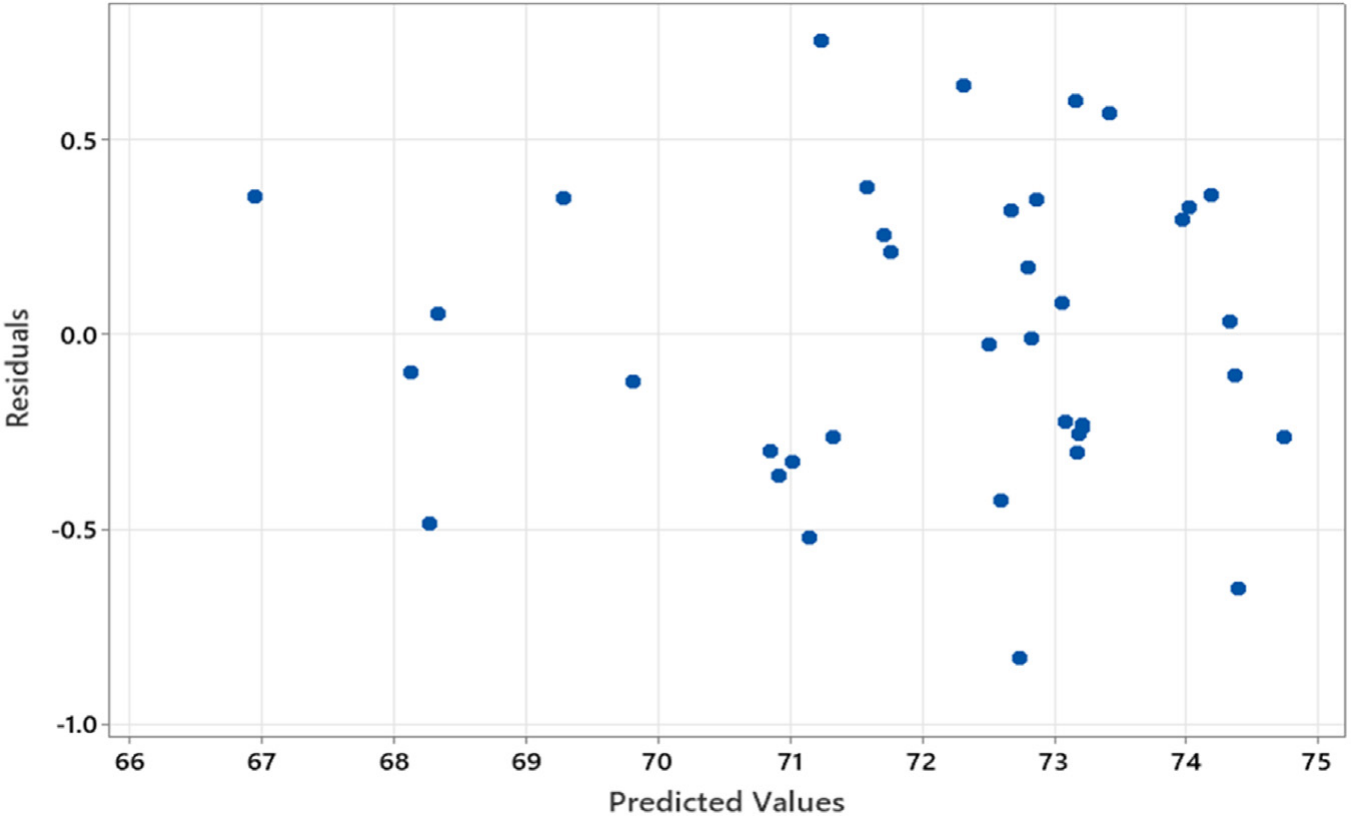
Residuals plot.

Fig. 2 shows the comparison between the actual and predicted values of the response variable. Based on Fig. 2, the predicted values follow the actual values of the response variable closely. This indicates that the model estimation fits well for predicting the response variable. Moreover, in term of efficiency of the model estimation, we have checked for the presence or absence of heteroscedasticity in the residuals of the model estimation. This can be seen using the residuals plot and the Glejser test, where the residuals plot is given in Fig. 3.

Fig. 3 shows the residuals plot of the model estimation with the predicted values, where the residual values are obtained by the difference between the actual and predicted values of the response variable. Based on Fig. 3, the residuals are randomly scattered without any clear pattern, indicating that the model estimation fits well and there is no evident heteroscedasticity. In addition to the graphical analysis, we also conducted a Glejser test. The results showed the simultaneous P−value of 0.738, which is greater than the 5 % significance level. Furthermore, at a 5 % significance level, no individual parameters were found to be significant when regressing the absolute errors against all predictor variables (including predictor variables for the cos functions). Therefore, based on the residuals plot and the Glejser test, we conclude that there is no heteroscedasticity present in the residuals of the model.

## Limitations

Not applicable.

## Ethics statements

The data used in this research is secondary data collected from the Badan Pusat Statistik (BPS) of East Java Province during 2022. This data is available upon request or can be accessed directly from the website of the Badan Pusat Statistik (BPS) of East Java Province at https://jatim.bps.go.id/.

## CRediT authorship contribution statement

**Mustain Ramli:** Conceptualization, Methodology, Software, Writing – original draft. **I Nyoman Budiantara:** Conceptualization, Methodology, Writing – review & editing, Validation, Supervision. **Vita Ratnasari:** Conceptualization, Methodology, Writing – review & editing, Validation, Supervision.

## Declaration of competing interest

The authors declare that they have no known competing financial interests or personal relationships that could have appeared to influence the work reported in this paper.

## Data Availability

Data will be made available on request.

## References

[1] Eubank R.L. (1999).

[2] Hardle W. (1990).

[3] Cizek P., Sadikoglu S. (2020). Robust nonparametric regression: a review. WIREs Comput. Stat..

[4] Kuter S., Akyurek Z., Weber G.W. (2016). Int. Arch. Photogramm. Remote Sens. Spatial Inf. Sci. XLII-2/W1.

[5] Ahmed S.E., Aydin D., Yilmaz E. (2020). Estimating the nonparametric regression function by using pade approximation based on total least squares. Numer. Funct. Anal. Optim..

[6] Putra R., Fadhlurrahman M.G. (2023). Determination of the best knot and bandwidth in geographically weighted truncated spline nonparametric regression using generalized cross validation. MethodsX.

[7] Aydin D., Guneri O.I., Fit A. (2016). Choice of bandwidth for nonparametric regression models using kernel smoothing: a simulation study. Int. J. Sci..

[8] Lee A.B., Izbicki R. (2016). A spectral series approach to high-dimensional nonparametric regression. Electron. J. Stat..

[9] Zhu T., Politis D.N. (2017). Kernel estimates of nonparametric function autoregression models and their bootstrap approximation. Electron. J. Stat..

[10] Bilodeau M. (1992). Fourier smoother and additive models. Canad. J. Stat..

[11] Canditiis D.D., Feis I.D. (2006). Pointwise convergence of Fourier regularization for smoothing data. J. Comput. Appl. Math..

[12] Pane R., Budiantara I.N., Zain I., Otok B.W. (2013). Fourier series estimator in nonparametric multivariable regression and its characteristics. Proc. Int. Conf. Appl. Stat..

[13] Mardianto M.F.F., Gunardi H.U. (2021). An analysis about Fourier series estimator in nonparametric regression for longitudinal data. Math. Stat..

[14] Srivani A., Arunkumar T., Ashok S.D. (2018). Fourier harmonic regression method for bearing condition monitoring using vibration measurements. Mater. Today.

[15] Steinerberger S. (2021). Wasserstein distance, Fourier series and applications. Monatsh. Math..

[16] Octavanny M.A.D., Budiantara I.N., Kuswanto H., Rahmawati D.P. (2021). Modeling of children ever born in Indonesia using Fourier series nonparametric regression. J. Phys. Conf. Ser..

[17] Zoumb P.A.W., Li X. (2023). Fourier regression model predicting train-bridge interactions under wind and wave actions. Struct. Infrastruct. Eng..

[18] Iriany A., Fernandes A.A.R. (2023). Hybrid Fourier series and smoothing spline path non-parametric estimation model. Front. Appl. Math. Stat..

[19] Ramli M., Budiantara I.N., Ratnasari V. (2023). A method for parameter hypothesis testing in nonparametric regression with Fourier series approach. MethodsX.

[20] Saputro D.R.S., Sukmayanti A., Widyaningsih P. (2019). The nonparametric regression model using Fourier series approximation and Penalized Least Squares (PLS) (case on data proverty in East Java). J. Phys. Conf. Ser..

[21] Prahutama A., Suparti T.W.U. (2018). Modelling Fourier regression for time series data- a case study: modelling inflation in foods sector in Indonesia. J. Phys. Conf. Ser..

[22] Zhang J.T. (2011). Statistical inferences for linear models with functional responses. Stat. Sin..

[23] Wei Z., Fan Y., Zhang J. (2016). Statistical inference on restricted regression models with partial distortion measurement errors. Braz. J. Probab. Stat..

[24] Guo X., Li R., Liu J., Zeng M. (2024). Reprint: statistical inference for linear mediation models with high-dimensional mediators and application to studying stock reaction to COVID-19 pandemic. J. Econom..

[25] Craven P., Wahba G. (1979). Smoothing noisy data with spline functions. Numer. Math..

[26] Fonseca M., Mexia J.T., Sinha B.K., Zmyslony R. (2015). Likelihood ratio tests in linear models with linear inequality restrictions on regression coefficients. REVSTAT-Stat. J..

[27] He Y., Jiang T., Wen J., Xu G. (2021). Likelihood ratio test in multivariate linear regression: from low to high dimension. Stat. Sin..

[28] Liu Y., Zhu J., Lin D.K.J. (2021). A generalized likelihood ratio test for monitoring profile data. J. Appl. Stat..

[29] Searle S.R. (1997).

[30] Casella G., Berger R.L. (2002).

